# Contrast tomography (CT) performed to detect nodal metastasis for high-risk cutaneous squamous cell carcinomas of the head and neck has a high negative predictive value but a poor positive predictive value

**DOI:** 10.1016/j.jdin.2023.07.004

**Published:** 2023-07-21

**Authors:** Kyle Rudningen, Kimberly A. Sable, Tiffany A. Glazer, Rong Hu, Michael R. Lasarev, Yaohui Gloria Xu

**Affiliations:** a Department of Dermatology, CentraCare, St. Cloud, Minnesota; b Department of Dermatology, University of Wisconsin School of Medicine and Public Health, Madison, Wisconsin; c Division of Otolaryngology-Head & Neck Surgery, UW Department of Surgery, Clinical Science Center, University of Wisconsin School of Medicine and Public Health, Madison, Wisconsin; d Department of Pathology and Laboratory Medicine, University of Wisconsin School of Medicine and Public Health, Madison, Wisconsin; e Department of Biostatistics and Medical Informatics, University of Wisconsin School of Medicine and Public Health, Madison, Wisconsin

**Keywords:** CT scan, head and neck, imaging, metastasis, predictive value, sensitivity, specificity, squamous cell carcinoma

*To the Editor:* In contrast to the considerable data on imaging for staging oropharyngeal head and neck squamous cell carcinoma (HNSCC), the accuracy of contrast tomography (CT) to detect nodal metastases in cutaneous squamous cell carcinoma (cSCC) is not established.^1^^,^^2^ We performed a retrospective study assessing sensitivity and specificity of CT to detect nodal metastasis among high-risk cSCC of the head and neck. Our reference standard (ie, “true”) result was the conclusion from nodal pathology sampling. This was compared to the result of CT studies performed prior to nodal sampling. Cases were identified using a combination of the relevant International Classification of Diseases (ICD 9/10) codes and nodal sampling Current Procedural Terminology (CPT) codes in the electronic medical record over a 10-year interval from July 1, 2011 to June 30, 2021. This resulted in a cohort of 38 patients with 40 tumors.

Nodal sampling type varied; however, most cases (34/40) received neck dissection (including selective or radical neck dissection) and/or parotidectomy. Neck dissection and/or parotidectomy were performed due to the estimated high risk of metastasis in these patients. This was based on the advanced average size of 5.5 cm or due to imaging or examination findings suggesting metastasis. In those who did not have neck dissection and/or parotidectomy, 3 patients had sentinel node biopsies, 2 had fine needle aspirations, and 1 case had nodal excision. All patients had CT prior to surgery with 37 out of 40 being performed with contrast. Information was collected on each tumor to better characterize the cohort (Table I).

**Table I:** Tumor characteristics and staging

Palpable adenopathy on exam	13/40
Average tumor size	5.5 cm
Recurrent	19/40
Perineural invasion	16/40
Lymphovascular invasion	6/40
Immunosuppressed status	10/40
AJCC8 Stage∗	
T1	1
T2	0
T3	33
T4a	1
T4b	5
BWH Stage†	
T1	1
T2a	9
T2b	24
T3	6

∗ American Joint Committee on Cancer Staging Manual, eighth Edition.^3^

† Brigham and Women's Hospital Tumor Classification System for Cutaneous Squamous Cell Carcinoma.^3^

The calculated sensitivity of imaging to detect nodal metastasis in our cohort was 90% [95% confidence interval: 70% to 97%] while specificity was 55% [34% to 74%]. Values were re-calculated after excluding 13 patients with palpable adenopathy due to concern of possibly overstating the sensitivity. Sensitivity then became more modest at 78% [45% to 94%] and specificity was similar at 61% [39% to 80%]. Based on our data and an estimated metastasis rate of 23.8% [14.8% to 38.4%] for T2b tumors as was found in a 2019 study by Dr Ruiz and colleagues,^3^ the positive predictive value of CT to detect metastasis in a T2b tumor without clinical adenopathy is 38% [20% to 62%] and the negative predictive value is 90% [72% to 97%] (Table II).

**Table II:** Results

Full cohort, *n* = 40	
# Of nodal metastases	20/40
Imaging positive∗	27/40
Sensitivity	90% [70-97%]
Specificity	55% [34-74%]
PPV	38% [26-60%]
NPV	95% [82-99%]
Patients without palpable adenopathy, *n* = 27	
#Of nodal metastases	9/27
Imaging positive∗	14/27
Sensitivity	78% [45-94%]
Specificity	61% [39-80%]
PPV	38% [20-62%]
NPV	90% [72-97%]

95% confidence intervals in brackets.

Predictive values calculated using our study’s sensitivity and specificity data and a pre-test probability of 23.8% [14.8% to 38.4%] risk of metastasis. This is the rate of metastasis in T2b tumors as reported by Dr Ruiz & colleagues.^3^

*NPV*, Negative predictive value; *PPV*, positive predictive value.

∗ Imaging positive for nodal metastasis.

We conclude that CT likely has some utility in ruling out nodal metastasis, however the false positive rate is high. We suspect the reason for the high false positive rate may be due to enlarged reactive nodes in response to the primary tumor. With such a high false positive rate, sampling of an enlarged node should be considered prior to proceeding with neck dissection.

Limitations to this study include retrospective design with small sample size as well as improvements in imaging technology over time. We chose nodal sampling as the reference standard as opposed to long-term followup as followup was often short and variable. This emulates the oropharyngeal SCC literature which predominantly uses radical neck dissection as the reference standard when evaluating imaging accuracy.^4^ Nodal sampling cannot detect all metastases and therefore the sensitivity may be overstated. Prospective data with long-term followup assessing multiple imaging modalities is warranted.

## Conflicts of interest

None disclosed.

## References

[1] Fox M., Brown M., Golda N. (2019). Nodal staging of high-risk cutaneous squamous cell carcinoma. J Am Acad Dermatol.

[2] Ruiz E.S., Karia P.S., Morgan F.C., Schmults C.D. (2017). The positive impact of radiologic imaging on high-stage cutaneous squamous cell carcinoma management. J Am Acad Dermatol.

[3] Ruiz E.S., Karia P.S., Besaw R., Schmults C.D. (2019). Performance of the American Joint Committee on Cancer staging manual, 8th edition vs the Brigham and Women's Hospital tumor classification system for cutaneous squamous cell carcinoma. JAMA Dermatol.

[4] Bree R., Takes R.P., Castelijns J.A. (2015). Advances in diagnostic modalities to detect occult lymph node metastases in head and neck squamous cell carcinoma. Head Neck.

